# Proteomic Analysis of Matched Formalin-Fixed, Paraffin-Embedded Specimens in Patients with Advanced Serous Ovarian Carcinoma

**DOI:** 10.3390/proteomes1030240

**Published:** 2013-10-17

**Authors:** Ashlee L. Smith, Mai Sun, Rohit Bhargava, Nicolas A. Stewart, Melanie S. Flint, William L. Bigbee, Thomas C. Krivak, Mary A. Strange, Kristine L. Cooper, Kristin K. Zorn

**Affiliations:** 1 Division of Gynecologic Oncology, Magee-Womens Hospital of UPMC, Pittsburgh, PA 15213, USA; E-Mails: tomkrivak@verizon.net (T.C.K.); kkzorn@aol.com (K.K.Z.); 2 Biomedical Mass Spectrometry Center for the Health Sciences, University of Pittsburgh, Pittsburgh, PA 15213, USA; E-Mails: sunm3@upmc.edu (M.S.); nas96@pitt.edu (N.A.S.); 3 Department of Pathology, University of Pittsburgh School of Medicine, Pittsburgh, PA 15213, USA; E-Mail: rbhargava@mail.magee.edu; 4 Departments of Pharmacology and Chemical Biology, University of Pittsburgh School of Medicine, Pittsburgh, PA 15213, USA; E-Mail: flintms@upmc.edu; 5 Women’s Cancer Research Center, Pittsburgh, PA 15213, USA; E-Mails: bigbeewl@upmc.edu (W.L.B); mstrange@mwri.magee.edu (M.A.S.); 6 University of Pittsburgh Cancer Institute, Pittsburgh, PA 15213, USA; E-Mail: cooperk3@upmc.edu; 7 Magee-Womens Research Institute, Pittsburgh, PA 15213, USA

**Keywords:** serous ovarian carcinoma, proteomics, laser capture microdissection

## Abstract

Objective: The biology of high grade serous ovarian carcinoma (HGSOC) is poorly understood. Little has been reported on intratumoral homogeneity or heterogeneity of primary HGSOC tumors and their metastases. We evaluated the global protein expression profiles of paired primary and metastatic HGSOC from formalin-fixed, paraffin-embedded (FFPE) tissue samples. Methods: After IRB approval, six patients with advanced HGSOC were identified with tumor in both ovaries at initial surgery. Laser capture microdissection (LCM) was used to extract tumor for protein digestion. Peptides were extracted and analyzed by reversed-phase liquid chromatography coupled to a linear ion trap mass spectrometer. Tandem mass spectra were searched against the UniProt human protein database. Differences in protein abundance between samples were assessed and analyzed by Ingenuity Pathway Analysis software. Immunohistochemistry (IHC) for select proteins from the original and an additional validation set of five patients was performed. Results: Unsupervised clustering of the abundance profiles placed the paired specimens adjacent to each other. IHC H-score analysis of the validation set revealed a strong correlation between paired samples for all proteins. For the similarly expressed proteins, the estimated correlation coefficients in two of three experimental samples and all validation samples were statistically significant (*p* < 0.05). The estimated correlation coefficients in the experimental sample proteins classified as differentially expressed were not statistically significant. Conclusion: A global proteomic screen of primary HGSOC tumors and their metastatic lesions identifies tumoral homogeneity and heterogeneity and provides preliminary insight into these protein profiles and the cellular pathways they constitute.

## 1. Introduction

Epithelial ovarian cancer (EOC) is the most lethal gynecologic malignancy [1]. The majority of patients with EOC present with metastatic lesions identified at primary surgery and experience recurrence. This type of clinical behavior affords researchers the opportunity to evaluate multiple tumor samples throughout a patient’s clinical course. 

The biology of high-grade serous ovarian carcinoma (HGSOC) and its metastases remains poorly understood [2,3]. Serous carcinomas are the most common subtype of EOC and are further divided into low-grade and high-grade based on molecular and morphologic features. Low-grade serous ovarian carcinoma is frequently associated with mutations in *BRAF* and *KRAS*, demonstrates mild to moderate nuclear atypia, and often expresses higher levels of estrogen receptors, progesterone receptors, and E-cadherin [3]. In contrast, HGSOC is defined by ubiquitous *TP53* mutations, defects in homologous DNA repair with chromosomal instability, and high cellular proliferation [2]. Given this genomic instability, the molecular features of HGSOC vary widely across tumors (intertumoral heterogeneity) but also within tumors (intratumoral heterogeneity) [2,4]. The role of tumoral heterogeneity in treatment response and recurrence patterns is not clearly defined.

Multiple high-throughput research techniques have been applied in studying HGSOC. Attempts to identify serum biomarkers, investigations of the histologic types, and genomic studies of metastatic lesions have been completed utilizing fresh-frozen samples [5,6,7,8,9]. However, extensive fresh-frozen tissue collections are rare. Formalin-fixed, paraffin-embedded (FFPE) tissue is the most common source for archived pathologic specimens. FFPE tissue collections have become important resources for studies of DNA, RNA, and more recently, intact proteins [10,11,12]. 

Proteomics involves the large-scale identification, characterization, and quantitation of proteins expressed in a tissue [13]. An advantage of proteomics is the ability to examine the biochemical and cellular phenotypic states, rather than genotypic expression patterns [14]. Mass spectrometry applications have been used to look for biomarkers in both the ascites and serum of EOC patients [15,16,17]. Additionally, studies have evaluated histologic subclassifications of EOC, characterized tumors by stage of disease, and analyzed samples of HGSOC for markers of chemosensitivity and chemoresistance [17,18,19,20,21]. Initially, proteomic studies utilized HGSOC tissue sources ranging from fresh-frozen specimens to ovarian cancer cell lines [13,22]. In 1998, Ikeda *et al.* described the extraction of intact protein from FFPE tissues [23]. Currently, faster and more efficient techniques have been developed to allow the extraction of full-length, non-degraded proteins from FFPE tissues [24,25,26]. Coupling abundant FFPE samples with laser capture microdissection (LCM) technology permits the dissection of particular areas of interest. Additionally, while access to fresh-frozen primary tumors and matched metastatic sites is often limited, matched FFPE tissues are readily available. 

To date, the proteomic profiles of primary and metastatic EOC at the time of initial debulking surgery have not been investigated. Proteomic analysis across different tissue sites (for example, omentum and ovary) can be complicated by the inability to distinguish the inherent background differences in protein expression between the sites from the differences that are due to tumor expression in each site. In this study, our goal was to evaluate the protein expression profiles via a global proteomic screening of HGSOC from FFPE samples of both ovaries, with the presumption that one side represents the primary tumor and the other side a metastatic site, therefore eliminating heterogeneity among different tissue types. Following identification, selected proteins involved with migration, cell adhesion, and metastasis were investigated using IHC to assess the correlation of findings.

## 2. Experimental

### 2.1. FFPE Collection, Processing for Proteomics

Following approval from the University of Pittsburgh IRB, FFPE samples from six patients with International Federation of Gynecology and Obstetrics Stages III or IV HGSOC and tumor present in both ovaries at the time of initial surgery were identified as the experimental set of patients (E-HGSOC). Five additional patients (V-HGSOC), matched for age and stage, were identified and used in addition to the original six patients for evaluation by IHC. All patients underwent initial debulking surgery at Magee-Womens Hospital of UPMC. Hematoxylin and Eosin (H&E) stained tissue specimens were evaluated by a gynecologic pathologist to confirm the diagnosis and presence of tumor in both ovaries. Tissue samples were cut by microtome to 10 µm sections and placed onto laser microdissection slides (Leica Microsystems, Inc., Bannockburn, IL, USA). Slides were lightly stained via standard H&E protocol immediately prior to completion of LCM. Cancer cells from defined regions of the ovaries were acquired by laser microdissection (Leica LMD 6000, Leica Microsystems, Inc.) and collected in 40 µL of purified water in RNase/DNase-free microcentrifuge tubes. Six million microns of tissue was the targeted amount to be collected. Samples were brought to 100 mM NH_4_HCO_3_, pH 8.4, 20% acetonitrile and incubated in a thermal cycler at 100 °C for 1 h, and thereafter, 65 °C for an additional 2 h. Samples were cooled to ambient temperature, followed by addition of 100 ng of modified porcine sequencing grade trypsin (Promega Corporation, Madison, WI, USA) and incubated for 16 h at 37 °C. Samples were vacuum-dried and desalted using PepClean desalting columns (Pierce, Rockford, IL, USA) according to the manufacturer’s protocol. Eluted peptides were vacuum-dried and stored at −80 °C. 

### 2.2. Liquid Chromatography Tandem Mass Spectrometry

Peptide digests were resuspended in 0.1% TFA and analyzed in triplicate by reversed-phase liquid chromatography (RPLC)-tandem mass spectrometry (MS/MS) using an Ultimate 3000 Nanoflow LC (Dionex Corporation, Sunnyvale, CA, USA) coupled online to an LTQ-Orbitrap XL (ThermoFisher Scientific, San Jose, CA, USA). Separations were performed using 75 µm ID × 360 µm OD × 20 cm-long fused silica capillary columns (Polymicro Technologies, Phoenix, AZ, USA), slurry-packed in house with 5 µm, 300 Å pore size C-18 silica-bonded stationary phase (Jupiter, Phenomenex, Torrance, CA, USA). Following sample injection onto the C-18 pre-column (Dionex), the column was washed for 3 min with mobile phase A (2% acetonitrile, 0.1% formic acid) at a flow rate of 30 µL/min. Peptides were eluted using a linear gradient of 0.33% mobile phase B (0.1% formic acid in acetonitrile)/min for 130 min, then to 95% B in an additional 15 min, all at a constant flow rate of 200 nL/min. Column washing was performed at 95% B for 15 min for all analysis, after which the column was re-equilibrated in mobile phase A prior to subsequent injections. The MS was operated in data-dependent MS/MS mode in which each full MS scan was collected in the Orbitrap, followed by seven MS/MS scans performed in the linear ion trap (LIT) where the seven most abundant peptide molecular ions were selected for collision-induced dissociation (CID), using a normalized collision energy of 35%. Data were collected over a precursor ion range of 375–1,800 *m/z* (R = 60,000 @ 400 *m/z*) and dynamic exclusion was enabled to minimize redundant selection of peptides previously selected for CID. 

### 2.3. Peptide Identification

Tandem mass spectra were searched against the UniProt human protein database (10/11 release) from the European Bioinformatics Institute, using SEQUEST (BioWorks, v3.2, ThermoScientific) [27]. Search criteria were set as follows: peptides were searched fully-tryptic with up to two missed cleavage sites, dynamic modification of methionine oxidation (15.9949 Da) and static modification of cysteine carbamidomethylation (57.0215 Da), precursor mass tolerance of 20 ppm and fragment ion tolerance of 0.5 Da. Identified peptides were further screened using the Peptide Prophet algorithm included in the Trans-Proteomic Pipeline software package (V4.3 rev.1) [28,29]. 

### 2.4. Spectral Count Analysis

The qualitative differences in protein abundance between samples were derived by summing the total CID events that resulted in a positively identified peptide for a given protein accession across all samples (spectral counting), as previously described [30]. Proteins included for analysis were filtered using the criteria that a minimum peptide count of two or greater for a given protein was present in at least four of the samples analyzed. The relative protein abundance levels between the right and left ovaries within each patient were assessed by fold changes. Proteins were compared between the right and left ovaries for each patient and classified by qualitative analysis of their spectral counts as proteins which were similarly expressed (less than a two-fold change) or differentially expressed (greater than or equal to a two-fold change) between the paired ovarian samples [31,32,33]. 

### 2.5. Ingenuity Pathways Analysis

Proteins identified (using UniProt accession numbers) as similarly expressed (without a two-fold change in at least four of the paired sets) or differentially expressed (with a two-fold change in at least four of the paired sets) were entered into IPA software (Ingenuity Systems, Redwood City, CA, USA) for pathway analysis. Functional analysis of these proteins was performed utilizing the “Core Analysis” function in IPA. 

### 2.6. Immunohistochemical Staining

Analysis of the IPA results, and review of established literature, resulted in the selection of the proteins evaluated via IHC. Antibodies selected included: CTNNB1 (BD Biosciences, San Jose, USA), CDC42 (Santa Cruz, Dallas, USA), PPP2R1A (Santa Cruz), ANXA1 (BD Biosciences), PHB (Sigma-Aldrich, St. Louis, USA), and PRDX1 (AbCam, Cambridge, UK) (Table S1). IHC was completed on the E-HGSOC and V-HGSOC paired sets. IHC of the 5-micrometer thick paraffin sections was performed on a Leica Bond immunostainer with Leica reagents (Buffalo Grove, IL, USA). Sections were deparaffinized and treated with heat-induced epitope retrieval solutions. The sections were incubated with the primary antibody, followed by the post primary antibody, and finally the Bond polymer. The sections were treated with a peroxide block and DAB. Sections stained for CDC42 were treated with an additional 5-min DAB enhancer step that was not included in the other antibody staining protocols. The sections were counterstained with hematoxylin. Washes separated each incubation step. Adequate immunoreactive tissue samples were used as positive controls. Negative controls consisted of omitting the primary antibody and replacing it with an antibody diluent solution.

The immunohistochemical staining was semi-quantified using the H-score method [34,35]. The H-score is given as the sum of the percent staining multiplied by an ordinal value corresponding to the intensity level (0 = none, 1 = weak, 2 = moderate, 3 = strong). With four intensity levels, the resulting score ranges from 0 (no staining in the tumor) to 300 (diffuse intense staining of the tumor). The following cellular areas were scored for each antibody: cytoplasm for PHB, CDC 42 and PRDX1; nucleus and cytoplasm for ANXA1 and PPP2R1A; both membranes and cytoplasm for CTNNB1. A Gynecologic Oncology Pathologist scored the IHC staining and was blinded to the specimen sources. 

Agreement of H-scores and percent staining between paired samples was estimated using Pearson’s correlation coefficient; 95% confidence intervals were reported for the experimental and validation sets separately.

## 3. Results

Patients meeting selection criteria for the E-HGOC and V-HGOC matched-paired sets were identified. All patients had stage III or IV disease. Most patients were Caucasian, with a mean age range of 45–50 years old (Table 1). 

**Table 1:** Patient demographics.

	E-HGSOC Specimens ^a,^*^,^**	V-HGSOC Specimens ^b,^*
	(n = 6)	(n = 5)
Age at Diagnosis (y)		
Age Range	45–50	45–50
Race		
Caucasian	5	5
African American	1	0
Stage		
IIIB	0	1
IIIC	4	4
IV	2	0

^a^ E-HGSOC: Experimental high-grade serous ovarian cancer specimens; ^b^ V-HGSOC: Validation high-grade serous ovarian cancer specimens; * All patients underwent primary debulking surgery between 1998 and 2012 at a single institution. All carcinomas evaluated were high-grade serous epithelial ovarian carcinomas; ** E-HGSOC specimens were analyzed via proteomic analysis and IHC, whereas the V-HGSOC specimens were analyzed by IHC only and used for validation of the proteomic findings.

**Figure 1:**
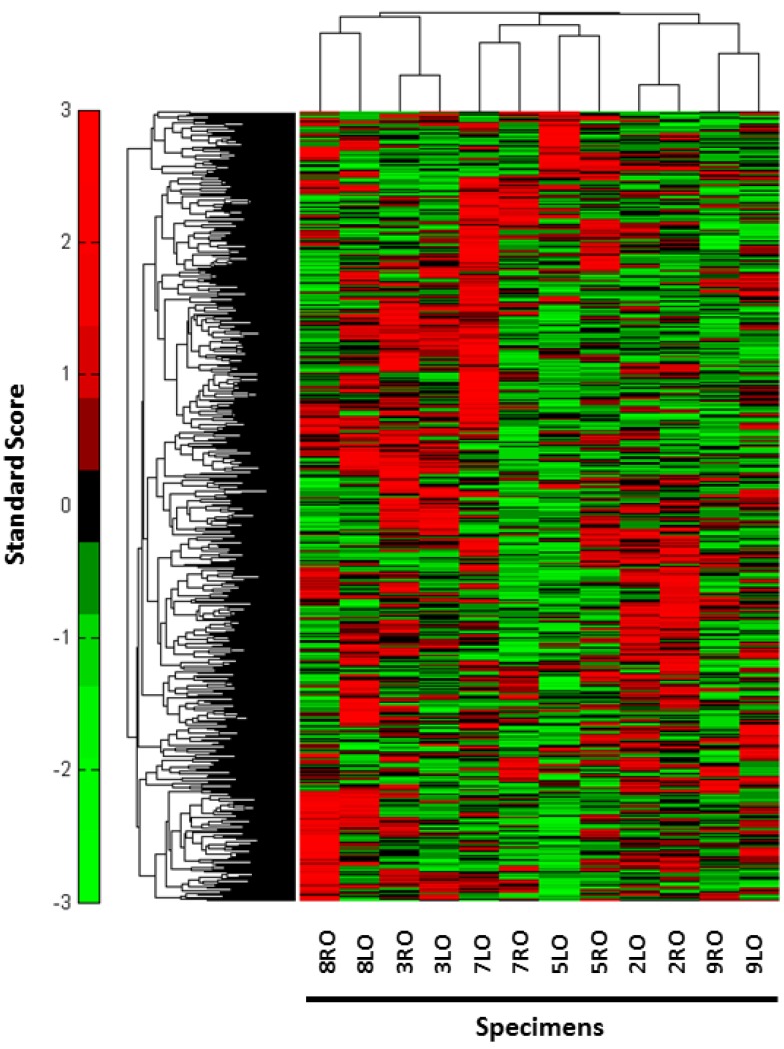
Unsupervised hierarchical cluster analysis. After initial filtering of the proteins generated by MS, unsupervised hierarchal clustering analysis was completed which highlights the similar and different protein abundance profiles across the samples when compared to each other. Six proteins including CTNNB1, CDC42, PPP2R1A, ANXA1, PHB, PRDX1 were then selected for validation. The red-green color scheme indicates the rank normalized abundance of a protein relative to its average value across all six patients. (MATLAB script, MathWorks®, Natick, MA, USA).

A total of six slides per disease site were prepared for LCM analysis. One to two slides from FFPE blocks of ovarian tissue yielded a mean of 7.11 micrograms from LCM. Complete results of the spectral counts obtained from MS can be found in the supplemental data (Table S2). After cursory filtering of the MS data described above, unsupervised hierarchical clustering of the protein abundance profiles consistently placed the original six paired specimens (E-HGOC) adjacent to each other, indicating that within each patient, gross aspects of tumoral homogeneity were observed (Figure 1). While globally there were similarities identified between the samples, areas of distinct heterogeneity were also noted. 

IPA for the proteins classified as similarly expressed yielded scores of 19 or higher for the top five networks. For proteins classified as differentially expressed, the top five networks had IPA scores of 13 or higher. Among the differentially expressed proteins, the most statistically significant network contained 35 interacting proteins from our original proteomic analysis. Top canonical pathways identified for similarly expressed proteins involved EIF2 signaling, gluconeogenesis/glycolysis, and regulation of eIF4 signaling. Canonical pathways identified most frequently among differentially expressed proteins included EIF2 signaling, acute phase response signaling, and aminoacyl-tRNA biosynthesis (Table S3A,B).

**Figure 2:**
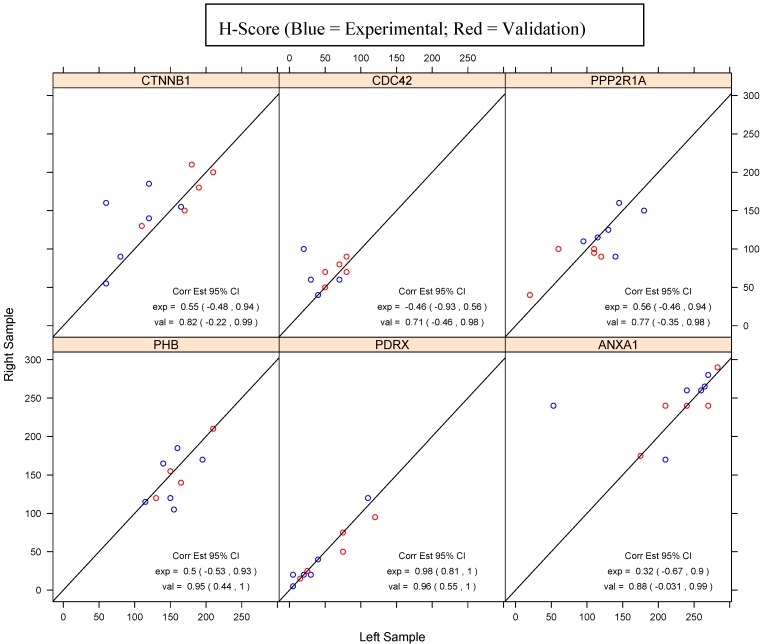
Correlation of paired samples H-Scores across proteins.

Six proteins noted on the IPA pathways, including CTNNB1, CDC42, PPP2R1A, ANXA1, PHB, PRDX1, were selected for IHC evaluation. Three proteins each were selected from the lists of similarly expressed proteins (ANXA1, PHB, PRDX1) and differentially expressed proteins (CTNNB1, CDC42, PPP2R1A). IHC staining was completed on the E-HGSOC and V-HGSOC paired samples (Table 2 and Table 3). The validation data set shows a strong correlation of the H-scores between the paired samples for all proteins, whereas the H-score correlation for the experimental set shows greater variation. The estimated correlation coefficients in the validation samples for the similarly expressed proteins and two of the three estimated correlation coefficients for the experimental sample proteins classified as similarly expressed were statistically significant at the 0.05 level (Figure 2). However, the estimated correlation coefficients in the experimental sample proteins classified as differentially expressed were not statistically significant. 

**Table 2:** Immunohistochemistry (IHC) table of results for antibodies representing proteins identified as having less than a two-fold change between paired samples.

	PHB		PDRX		ANXA1	
Sample	H-score	% staining of cells	H-score	% staining of cells	H-score	% staining of cells
E-HGSOC 2R	115	70	120	80	280	95
E-HGSOC 2L	115	70	110	80	270	95
E-HGSOC 3R	120	70	20	20	260	90
E-HGSOC 3L	150	75	20	20	240	90
E-HGSOC 5R	170	90	40	30	240	95
E-HGSOC 5L	195	95	40	30	53	20
E-HGSOC 7R	185	95	20	20	265	95
E-HGSOC 7L	160	85	5	5	265	95
E-HGSOC 8R	105	60	5	5	260	100
E-HGSOC 8L	155	75	5	5	260	100
E-HGSOC 9R	165	90	20	20	170	60
E-HGSOC 9L	140	85	30	30	210	80
V-HGSOC 1R	210	100	25	25	240	80
V-HGSOC 1L	210	100	25	25	270	90
V-HGSOC 2R	140	90	95	60	240	80
V-HGSOC 2L	165	90	120	70	210	70
V-HGSOC 3R	155	95	50	40	240	80
V-HGSOC 3L	150	80	75	50	240	90
V-HGSOC 4R	120	70	15	10	175	60
V-HGSOC 4L	130	90	15	15	175	60
V-HGSOC 5R	120	90	75	50	290	100
V-HGSOC 5L	130	90	75	50	283	98

E-HGSOC: experimental high grade serous ovarian carcinoma samples; V-HGSOC: validation high grade serous ovarian carcinoma samples; The H-score is given as the sum of the percent staining multiplied by an ordinal value corresponding to the intensity level (0 = none, 1 = weak, 2 = moderate, 3 = strong). With four intensity levels, the resulting score ranges from 0 (no staining in the tumor) to 300 (diffuse intense staining of the tumor).

**Table 3:** IHC table of results for antibodies representing proteins identified as having a two-fold or greater change between paired samples (see the note of Table 2).

	CTNNB1		CDC42		PPP2R1A	
Sample	H-score	% staining of cells	H-score	% staining of cells	H-score	% staining of cells
E-HGSOC 2R	55	50	60	60	150	90
E-HGSOC 2L	60	60	30	30	180	95
E-HGSOC 3R	155	80	60	60	110	60
E-HGSOC 3L	165	85	70	70	95	60
E-HGSOC 5R	160	100	100	80	160	90
E-HGSOC 5L	60	40	20	20	145	80
E-HGSOC 7R	140	85	60	60	125	90
E-HGSOC 7L	120	80	70	70	130	80
E-HGSOC 8R	90	60	50	50	90	70
E-HGSOC 8L	80	50	50	50	140	80
E-HGSOC 9R	185	95	40	30	115	70
E-HGSOC 9L	120	85	40	30	115	75
V-HGSOC 1R	150	100	90	90	100	70
V-HGSOC 1L	170	100	80	80	110	60
V-HGSOC 2R	180	100	80	80	95	75
V-HGSOC 2L	190	95	70	70	110	80
V-HGSOC 3R	130	80	70	70	100	70
V-HGSOC 3L	110	80	80	80	60	50
V-HGSOC 4R	200	100	70	70	90	70
V-HGSOC 4L	210	100	50	50	120	70
V-HGSOC 5R	210	100	50	50	40	40
V-HGSOC 5L	180	100	50	50	20	20

## 4. Discussion

Both intratumoral and intertumoral heterogeneity may play a significant role in tumor evolution, adaptation, and recurrence. This tumor variation can limit clinicians, who typically depend on the results of single tumor biopsies as they attempt to individualize treatment [4]. Better understanding of the biology of metastasis is critical, since we only rarely cure EOC when metastasis is present at diagnosis and are not able to cure recurrent EOC. The inherent complexity of genomic alterations between primary tumor and its metastases, coupled with numerous interactions between tumor and stromal cells, represent fundamental challenges in the quest to understand and control metastatic disease [14]. Comparisons of primary breast carcinomas with their corresponding metastatic brain lesions have demonstrated significant differences in the numbers of mutations found in a subset of cancer cells present in the primary lesion as compared to the metastatic site [36]. Genetic sequencing has now identified additional driver mutations in a subset of organ-specific metastases from pancreatic carcinoma that are not identified in the primary lesions [37]. These examples of key differences in the molecular biology of primary tumors and their metastatic lesions signal the potential difficulty of studying primary tumors as surrogates for what actually kills patients, their sites of metastatic spread. 

Herein, we elected to study primary and metastatic HGSOC within ovarian tissue. We analyzed six patients with advanced HGSOC with tumor present in both ovaries at their initial surgery to investigate the similarities and differences between these tumors. Ovarian tissue was used for comparison in an attempt to eliminate inherent protein expression differences among tissue sites, such as ovary, omentum, and lymph nodes. We integrated the techniques of LCM of FFPE specimens and mass spectrometry via discovery proteomics to assess the global proteomic profiles of these samples for similarly and differentially expressed proteins. 

Proteins selected for evaluation via IHC were extrapolated from both the proteomic data obtained and their relevance in the literature, particularly those related to the concepts of heterogeneity and metastasis. *PRDX1* has been identified as overexpressed in various subtypes of ovarian carcinoma and is implicated in the promotion and support of transformed tumor cells [38]. In prostate carcinoma specifically, PRDX family members are differentially expressed throughout various aspects of the tumor and have been suggested as a key family of proteins related to the disease process and response to treatment [39]. Underexpression of classes of annexins has been associated with chemoresistant disease and subsequently poor overall survival in ovarian carcinoma [18]. *CDC42* and *CTNNB1* are associated with cell-to-cell signaling and interaction, cellular assembly and organization, and tissue development. Both have demonstrated importance in the origination and continued propagation of disease in ovarian carcinoma and other solid tumors [40,41,42]. PPP2R1A has been identified as a key protein in the subclassifications of both serous endometrial and ovarian carcinomas, as well as clear cell carcinomas of the ovary. Mutations of this protein are linked to loss of negative regulation of cellular proliferation and loss of potential tumor suppression [43,44]. 

We recognize the small sample size for the original patients selected and the number of control cases as a limitation of our current study. However, this pilot project confirmed the feasibility of using LCM of FFPE tissue for discovery proteomics. Analysis with IHC did not correlate exactly with the proteomic findings in all cases. Perhaps our selected fold changes between samples were not significant enough to be detected using IHC methodology. Alternatively, spectral counting may not accurately quantitate these fold changes. Having completed the pilot attempt, additional patients can be studied. Using expanded results, further mechanistic avenues can be investigated to expand on the above noted concepts. 

The complex biology of EOC remains a challenge for researchers trying to identify biomarkers for early diagnosis and clinicians treating patients with advanced disease. The advanced stage at disease presentation, varied individual responses to surgery and chemotherapy, and poor overall survival open many avenues for exploration in an attempt to better understand this disease. Identification of similarities and differences between primary tumors and their metastatic lesions on multiple structural levels may provide further insight into this challenging disease, and ultimately further our efforts to discovering a cure. 

## 5. Conclusions

Proteomic analysis of HGSOC specimens from FFPE tissue is feasible, opening the door to further study with these samples that are well-annotated for clinical outcomes of interest. A global proteomic screen of primary HGSOC tumors and their metastatic lesions identifies tumoral homogeneity and heterogeneity, and provides preliminary insight into these protein profiles and the cellular pathways they constitute.

## Supplementary Materials

Supplementary File 1
