# Supplementary material for: Proteomic Analysis of Matched Formalin-Fixed, Paraffin-Embedded Specimens in Patients with Advanced Serous Ovarian Carcinoma
Source: Proteomes. 2013 Oct 17;1(3):240–53. doi: 10.3390/proteomes1030240 (PMC5302701; doi:10.3390/proteomes1030240)
Supplement: Supplementary File 1 [file proteomes-01-00240-s001.zip › proteomes-39065-Supplementary.docx]

Supplementary Material for

Proteomic Analysis of Matched Formalin-Fixed,
Paraffin-Embedded Specimens in Patients with Advanced Serous Ovarian Carcinoma

**Table S1.** Immunohistochemical antibodies and processing conditions.

| Antibody | Provider | Product Number |  | Dilution | Antigen Retrieval | Bond Protocol |
| --- | --- | --- | --- | --- | --- | --- |
| ANXA1 | BD Biosciences | 610067 | Monoclonal | 1:750 | ER2 for 20 min | 15/8/8 |
| CDC42 | Santa Cruz | sc-8401 | Monoclonal | 1:100 | ER2 for 40 min | 15/8/8 DAB Enhancer |
| CTNNB1 | BD Biosciences | 610153 | Monoclonal | 1:500 | ER1 for 30 min | 15/8/8 |
| PHB | Sigma-Aldrich | HPA003280 | Polyclonal | 1:300 | ER1 for 20 min | 30/8/8 |
| PPP2R1A | Santa Cruz | sc-13600 | Monoclonal | 1:50 | ER2 for 40 min | 30/8/8 |
| PRDX1 | AbCam | ab15571 | Polyclonal | 1:600 | ER1 for 30 min | 30/8/8 |

**Table S2.** Global, filtered proteomic data for the matched specimens collected (see attached Excel File).This table identifies the spectral count values obtained for the samples analyzed for the six experimental high grade serous ovarian carcinoma patients studied. The values from right and left ovary for each patient are further identified. The first two columns contain each protein with its accession number. The second and third tabs identify the proteins that were identified most frequently as similar or dissimilar between the samples from analysis of their spectral count change.

**Table S3.** IPA analysis reports generated for similarly or differentially expressed focus molecules. (**A**) IPA analysis report of proteins identified as having less than a two-fold change between paired samples.

| **Top Networks** | | |  |  |
| --- | --- | --- | --- | --- |
| **ID** | **Associated Network Functions** | **Score** | **Molecular and Cellular Functions** | **Number of Molecules** |
| 1 | RNA Post-Transcriptional Modification, DNA Replication, Recombination and Repair, Connective Tissue Disorders | 45 | EIF2 Signaling | 0.159 |
| 2 | Cancer, Reproductive System Disease, Cell-To-Cell Signaling and Interaction | 36 | Glycolysis/Gluconeogenesis | 0.203 |
| 3 | Dermatologic Diseases and Conditions, Immunologic Disease, and Inflammatory Disease | 32 | Citrate Cycle | 0.357 |
| 4 | Nucleic Acid Metabolism, Small Molecule Biochemistry, RNA Post-Transcriptional Modification | 21 | Regulation of eIF4 and p70S6K Signaling | 0.119 |
| 5 | Drug Metabolism, Endocrine System Development and Function, Lipid Metabolism | 19 | Pyruvate Metabolism | 0.188 |

**Table S3.** *Cont*.

**(B**) IPA analysis report of proteins identified as having a two-fold or greater change between paired samples.

| **Top Networks** | | |  |  |
| --- | --- | --- | --- | --- |
| **ID** | **Associated Network Functions** | **Score** | **Top Canonical Pathways** | **Ratio** |
| 1 | Cell-To-Cell Signalling and Interaction, Cellular Assembly and Organization, Tissue Development | 39 | EIF2 Signaling | 0.066 |
| 2 | Embryonic Development, Organismal Development, Tissue Development | 19 | Methane Metabolism | 0.267 |
| 3 | Cancer, Cell Cycle, Hereditary Disorders | 15 | Aminoacyl-tRNA Biosynthesis | 0.152 |
| 4 | Gastrointestinal Disease, Inflammatory Disease, Lipid Metabolism | 15 | Acute Phase Response Signaling | 0.058 |
| 5 | Carbohydrate Metabolism, Small Molecule Biochemistry, Cell Signalling | 15 | Glutamate Metabolism | 0.132 |
